# Characterization of the Rifamycin-Degrading Monooxygenase From Rifamycin Producers Implicating Its Involvement in Saliniketal Biosynthesis

**DOI:** 10.3389/fmicb.2020.00971

**Published:** 2020-06-03

**Authors:** Xiao-Fu Zheng, Xin-Qiang Liu, Shu-Ya Peng, Qiang Zhou, Bin Xu, Hua Yuan, Gong-Li Tang

**Affiliations:** ^1^Department of Chemistry, College of Sciences, Shanghai University, Shanghai, China; ^2^CAS-Key Laboratory of Synthetic Biology, Shanghai Institute of Plant Physiology and Ecology, University of Chinese Academy of Sciences, Chinese Academy of Sciences, Shanghai, China; ^3^Institute of Microbial Pharmaceuticals, College of Life and Health Sciences, Northeastern University, Shenyang, China; ^4^The Research Center of Chiral Drugs, Innovation Research Institute of Traditional Chinese Medicine, Shanghai University of Traditional Chinese Medicine, Shanghai, China; ^5^College of Life Sciences, Shanghai Normal University, Shanghai, China; ^6^State Key Laboratory of Bio-organic and Natural Products Chemistry, Shanghai Institute of Organic Chemistry, Chinese Academy of Sciences, Shanghai, China

**Keywords:** rifamycin degradation, Rox enzyme, flavin monooxygenase, self-resistance, saliniketal

## Abstract

Rifamycin derivatives, such as rifampicin, have potent antibiotic activity and have long been used in the clinic as mainstay components for the treatment of tuberculosis, leprosy, and AIDS-associated mycobacterial infections. However, the extensive usage of these antibiotics has resulted in the rapid development of bacterial resistance. The resistance mechanisms mainly include mutations of the rifamycin target RNA polymerase of bacteria and enzymatic modifications of rifamycin antibiotics. One modification is the recently characterized rifamycin degradation catalyzed by Rox enzymes, which belong to the widely occurring flavin monooxygenases. Intriguingly, our recent sequence analysis revealed the rifamycin producers also encode Rox homologs that are not yet characterized. In this work, we expanded the study of the Rox-catalyzed rifamycin degradation. We first showed that the Rox proteins from rifamycin producers have the enzymatic rifamycin SV-degrading activity. Then we used the structurally diverse rifamycin compounds rifampicin and 16-demethylrifamycin W to probe the substrate scope and found that they each have a slightly different substrate scope. Finally, we demonstrated that Rox proteins can also catalyze the transformation of 16-demethylsalinisporamycin to 16-demethylsaliniketal A. Since 16-demethylsalinisporamycin and 16-demethylsaliniketal A are the counterpart analogs of salinisporamycin and saliniketal A, our biochemical findings not only uncover a previously uncharacterized self-resistance mechanism in the rifamycin producers, but also bridge the gap between the biosynthesis of the potential antitumor compound saliniketal A.

## Introduction

**FIGURE 1:**
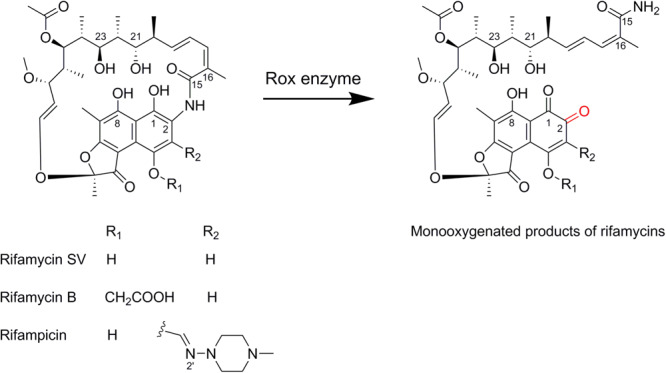
Rox enzyme-catalyzed degradation of Rif antibiotics.

Rifamycins (Rifs, Figure 1) are broad-spectrum antibiotics with potent antibiotic activity against both Gram-positive and Gram-negative bacteria (Floss and Yu, 2005). Their semi-synthetic derivatives, such as rifampicin, have long been mainstay components for the treatment of tuberculosis, leprosy, and AIDS-associated mycobacterial infections (Floss and Yu, 2005). Rif antibiotics target bacterial DNA-dependent RNA polymerases (RNAPs), and thus inhibit RNA transcription (Campbell et al., 2001). The crystal structure determination of the *Thermus aquaticus* core RNAP complexed with rifampicin revealed a pocket for Rif binding to the RNAP β subunit (RpoB) deep within the DNA/RNA channel (Campbell et al., 2001). For example, the four hydroxyl groups of Rif (at C-1, C-8, C-21, and C-23) form critical hydrogen bonds with RpoB. This strong interaction indicates that the antibiotic can effectively block the exit path for the newly-synthesized RNA (Campbell et al., 2001). However, the extensive usage of these antibiotics has resulted in the rapid development of bacterial resistance, and thus this class of antibiotics are commonly used in drug combinations (e.g., with isoniazid) and are restricted in treating tuberculosis or clinical emergencies so as to guarantee a relatively long lifespan of utility (Floss and Yu, 2005).

The mode of action predicts the antibiotic resistance mechanisms. Indeed, the majority of mutations to Rif resistance have been mapped to the *rpoB* gene in *Escherichia coli* and *Mycobacterium tuberculosis*, as well as other microbes examined (Floss and Yu, 2005). Moreover, the *rpoB* gene mutation also occurs in the Rif producer *Amycolatopsis mediterranei* (Floss and Yu, 2005). On the other hand, enzyme-catalyzed modifications of rifamycin antibiotics have been widely identified in environmental and pathogenic bacteria. Known enzymatic modifications mainly include the ADP-ribosylation on the hydroxyl group of C-23 catalyzed by Arr (Baysarowich et al., 2008), glycosylation on the hydroxyl group of C-23 by Rgt (Spanogiannopoulos et al., 2012), phosphorylation on the hydroxyl group of C-21 by Rph (Spanogiannopoulos et al., 2014), and Rif linearization (i.e., degradation) by Rox (Koteva et al., 2018). Consistent with the critical hydroxyl groups of C-21 and C-23 for Rif interactions with RpoB, the former three chemical group transfer modifications inevitably lead to its significantly low binding affinity. Altogether, these genetic elements constitute the Rif resistome, which negatively affects the expanded use of this class of antibiotics.

The recently characterized Rox enzymes belong to group A flavin monooxygenases, members of which have been widely distributed in the (poly)aromatic compound degradation and natural product biosynthesis (Huijbers et al., 2014). The Rox gene sequence was first identified from a genomic library of *Rhodococcus equi* known to be responsible for Rif decolorization (i.e., degradation), thus conferring increased resistance to rifampicin (Andersen et al., 1997). Later, a Rox homolog was characterized from *Nocardia farcinica* to catalyze rifampicin 2’-*N*-hydroxylation, which was then proposed to be responsible for the decolorization (Hoshino et al., 2010; Liu et al., 2016). Recently, however, Koteva et al. (2018) characterized a Rox homolog from *Streptomyces venezuelae* and revised that both the *S. venezuelae* Rox and *N. farcinica* Rox actually catalyze the C-2 hydroxylation and thereafter linearization of the Rif antibiotics (Figure 1). Indeed, this Rif-degrading mechanism is also consistent with the general function of group A flavin monooxygenases that hydroxylate phenolic compounds (Huijbers et al., 2014).

**FIGURE 2:**
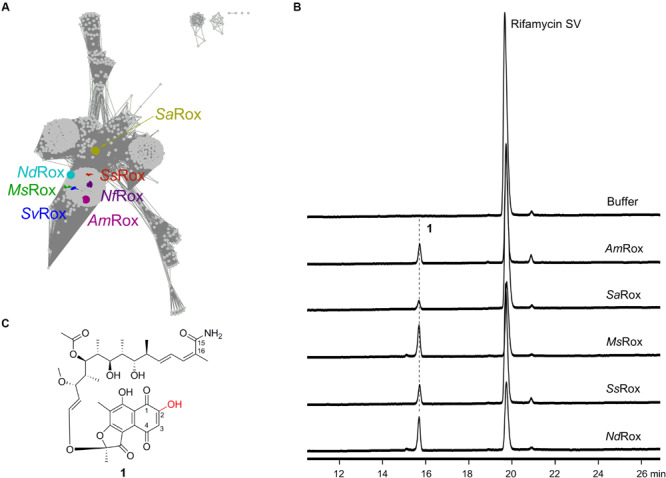
Characterization of the Rox homologs from Rif producers as well as other bacteria. **(A)** Sequence similarity network analysis of Rox homologs. The color-labeled proteins include *Am*Rox from the Rif SV producer *A. mediterranei* U32, *Ms*Rox from the 16-demethylrifamycin producer *Micromonospora* sp. TP-A0468, *Nd*Rox from *N*. *dokdonensis*, *Nf*Rox from *N. farcinica*, *Sa*Rox from the Rif SV producer *S*. *arenicola*, *Ss*Rox from the yatakemycin producer *Streptomyces* sp. TP-A0356, and *Sv*Rox from *S. venezuelae* ATCC 10712. These sequences show identities 66, 67, 61, 100, 49, 76, and 74% with *Nf*Rox, respectively. **(B)** HPLC analysis of the enzymatic products for the selected Rox homologs using Rif SV as substrate. The detection wavelength is 445 nm. **(C)** Structure of the enzymatic product **1**.

Our recent sequence analysis using the *N. farcinica* Rox sequence as query revealed that Rox homologs are conserved and widely distributed in environmental and pathogenic bacteria (Figure 2A). Intriguingly, the Rif producers (e.g., *A. mediterranei*, *Salinispora arenicola*, and *Micromonospora* sp. TP-A0468) also encode Rox homologs, but their functions are not yet determined. In this study, we first demonstrate that the Rox proteins from the Rif producers have the enzymatic Rif SV-degrading activity, which indicates that these proteins possess the ability to confer increased self-resistance to Rif antibiotics. Next, we find that members of the Rox proteins have a slightly different substrate scope by assessing structurally diverse Rif compounds. Finally, we prove that Rox proteins can also catalyze the transformation of 16-demethylsalinisporamycin to 16-demethylsaliniketal A. 16-Demethylsaliniketal A is a shunt product of the 16-demethylrifamycin producer *Micromonospora* sp. TP-A0468, which is a highly similar analog of the potential antitumor compound saliniketal A isolated from *S*. *arenicola* that inhibits the ornithine decarboxylase induction (Williams et al., 2007). Therefore, our biochemical findings not only establish that the Rif producers encode the Rif-degrading monooxygenase for potential self-resistance, but also bridge the gap of saliniketal biosynthesis.

## Results

### Characterization of the Rox Proteins From Rifamycin Producers as the Rif SV-Degrading Monooxygenase

In 2018, Koteva et al. (2018) identified a Rox homolog from *S. venezuelae* via the conserved rifampicin-associated element and then used the semi-synthetic Rif derivatives rifampicin and rifaximin as substrates to assess enzymatic activities (Spanogiannopoulos et al., 2014). The intensive 1D and 2D nuclear magnetic resonance (NMR) analyses of the reaction products unambiguously demonstrated that the Rox proteins from both *S. venezuelae* (*Sv*Rox) and *N. farcinica* (*Nf*Rox) can catalyze the linearization of the Rif antibiotics (Figure 1). In order to mine other functions of Rox proteins, we used the *Nf*Rox sequence as query and retrieved 4,886 Rox homologous sequences from the NCBI GenBank database as of November 2019 (Figure 2A). Among these homologs, the most distal sequence (from *Subtercola boreus*) shows 40% identity with *Nf*Rox. Intriguingly, the Rox homologs are also occurring in the Rif producers, such as *Am*Rox from the Rif SV producer *A. mediterranei* U32, *Sa*Rox from the Rif SV producer *S*. *arenicola*, and *Ms*Rox from the 16-demethylrifamycin producer *Micromonospora* sp. TP-A0468, all of which are not yet characterized.

In order to probe their functions, we chose *Am*Rox, *Sa*Rox, and *Ms*Rox, as well as two other Rox homologs: *Ss*Rox from *Streptomyces* sp. TP-A0356 (producer of yatakemycin) (Komaki et al., 2015) and *Nd*Rox from *Nocardioides dokdonensis* (Kwak et al., 2017) to test the Rif-degrading activity (Figure 2A). Their encoding gene sequences were obtained by either direct PCR amplifications or chemical synthesis (Supplementary Table S1). All five proteins were successfully expressed and purified from *E. coli* BL21(DE3) (Supplementary Figure S1). The isolated proteins showed the yellow color characteristic of bound FAD (Liu et al., 2016). For the enzymatic assays, Rif SV was first used as a substrate to assess the monooxygenation function. Based on the high-performance liquid chromatography (HPLC) analysis of the reaction assays, we found that all our selected proteins had the ability to transform Rif SV to a new compound **1** (Figure 2B), while the boiled enzymes did not. This new compound showed an ion peak at *m/z* 714.3118 [M + H]^+^ analyzed by the high resolution mass spectrometry (HR-MS; Supplementary Figure S2), indicating the molecular formula of C_37_H_47_NO_13_ (calculated for [M + H]^+^ as 714.3120). Therefore, this result suggests that **1** is likely to be the expected monooxygenation product of Rif SV (Figure 2C and Supplementary Figure S3).

To fully elucidate the structure of **1**, we performed large-scale enzymatic reactions so as to accumulate a sufficient amount for intensive NMR analyses. Based on the HMBC data (Supplementary Figure S4), we observed the expected correlation signals for the compound **1** that has an amino group at C-15 and a hydroxyl group at C-2: (1) the two proton signals (δ = 7.09 and 7.26) correlate with C-15 and C-16, which suggests that they belong to the amino group at C-15; and (2) the proton signal (δ = 15.15) correlates with C-2 and C-3, indicating a hydroxyl group at C-2. These two lines of evidence are fully consistent with the Rox-mediated linearization and lead us to conclude that the chemical structure of **1** is indeed the degradation product of Rif SV (Figure 2C and Supplementary Figure S4). Collectively, these results suggest that Rox enzymes are widely distributed and that all our five selected Rox proteins can degrade Rif SV.

### Substrate Scope Analysis of the Selected Rox Enzymes

**FIGURE 3:**
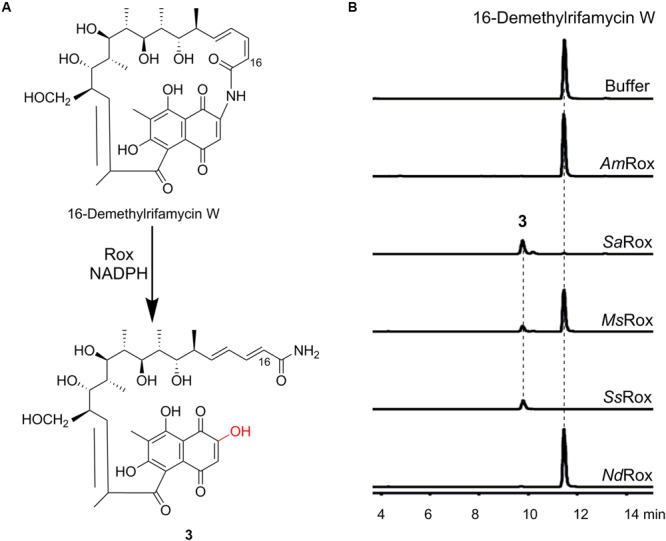
Enzymatic assays of 16-demethylrifamycin W. **(A)** Rox-catalyzed degradation of 16-demethylrifamycin W. **(B)** HPLC-MS analysis (base peak chromatograms) of the enzymatic products for the selected Rox homologs. The ion peaks of 16-demethylrifamycin W and the product **3** were extracted.

Next, we used two other structurally diverse substrates, rifampicin and 16-demethylrifamycin W, to assess the substrate scope of our selected Rox enzymes. Of the two compounds, 16-demethylrifamycin W was discovered from *Micromonospora* sp. TP-A0468 that originally biosynthesizes the antitumor polyketide kosinostatin by a genome mining strategy in our group (Zhou et al., 2019). This compound is a biosynthetic intermediate for 16-demethylrifamycin S biosynthesis, and like rifamycin W, the ansa chain does not go through the multiple oxidative rearrangement reactions (Zhou et al., 2019; Figure 3A and Supplementary Figure S5).

For the enzymatic assays of rifampicin, only *Ss*Rox can efficiently transform it to a new compound **2**, while the other enzymes have poor or no activity (Supplementary Figure S6). Subsequent HR-MS analysis revealed **2** with an ion peak at *m/z* 839.4071 [M + H]^+^, corresponding to a molecular formula of C_43_H_58_N_4_O_13_, which agrees well with the monooxygenation reaction (Supplementary Figure S6). Likewise, for the substrate 16-demethylrifamycin W, we found that *Sa*Rox, *Ms*Rox, and *Ss*Rox can accept 16-demethylrifamycin W and convert it to a new compound **3** (Figure 3B). And HR-MS analysis of **3** showed an ion peak at *m/z* 660.3012 [M + H]^+^, consistent with the molecular formula of C_34_H_45_NO_12_ (Supplementary Figure S7). This result also suggests the enzymatic monooxygenation reaction. Altogether, our biochemical results unambiguously demonstrate that Rox proteins have the ability to recognize structurally diverse rifamycin compounds, although they have a slightly different substrate scope.

### Assignment of Rox as the Missing Saliniketal Biosynthetic Enzyme

Saliniketals A and B are bicyclic polyketides isolated from *S*. *arenicola*, which can inhibit the ornithine decarboxylase induction that is an important anticancer target (Williams et al., 2007). It was demonstrated by Wilson et al. (2010) that the two compounds have the same biosynthetic origin with Rif SV, and they also showed *in vivo* that it’s the same cytochrome P450 encoded by *sare1259* in the Rif SV biosynthetic gene cluster that converts the intermediate 34a-deoxyrifamycin W to salinisporamycin via multiple oxidative rearrangement reactions (Matsuda et al., 2009; Supplementary Figure S5). However, the subsequent transformation of salinisporamycin to saliniketal A remains obscure.

**FIGURE 4:**
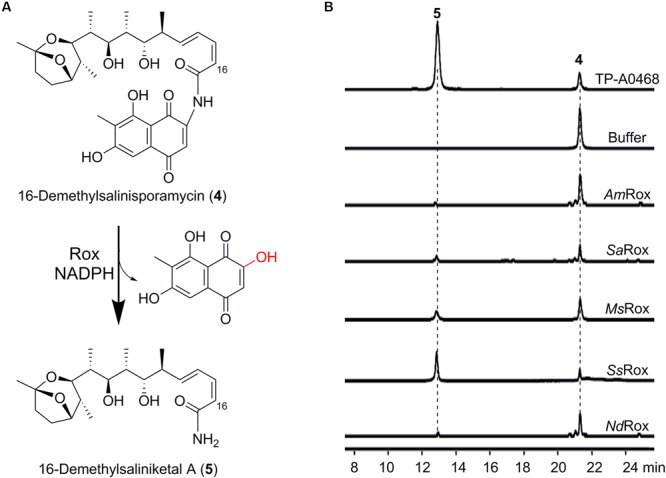
Enzymatic assays of 16-demethylsalinisporamycin. **(A)** Rox-catalyzed degradation of 16-demethylsalinisporamycin. **(B)** HPLC-MS analysis (base peak chromatograms) of the fermentation products of the Δ*kst* strain of *Micromonospora* sp. TP-A0468 and enzymatic products of the selected Rox homologs. The ion peaks of 16-demethylsalinisporamycin and 16-demethylsaliniketal A were extracted.

During our biosynthetic studies of 16-demethylrifamycins, we observed two compounds (**4** and **5**) corresponding to 16-demethylsalinisporamycin and 16-demethylsaliniketal A, respectively, in the fermentation broth of *Micromonospora* sp. TP-A0468 on the basis of HPLC-MS analysis (Figure 4B). To elucidate the structure of **4**, we carried out large-scale fermentation and accumulated a sufficient amount for its structural characterization. Subsequent HR-MS and NMR analyses confirmed that the compound **4** is the expected 16-demethylsalinisporamycin (Figure 4A and Supplementary Figures S8, S9).

Considering that Rox enzymes can recognize structurally diverse Rif compounds, we wonder whether Rox can catalyze the transformation of (16-demethyl)salinisporamycin to (16-demethyl)saliniketal A. To test this hypothesis, we next used the available compound **4** as substrate to probe the enzymatic activity. Fortunately, based on the HPLC-MS analysis we detected a new peak, which has the same retention time as the compound **5** observed in the fermentation broth of the Δ*kst* strain of *Micromonospora* sp. TP-A0468 (Figure 4B). Subsequent HPLC-HR-MS analysis of the reaction products showed **5** with an ion peak at *m/z* 382.2588 [M + H]^+^ (Supplementary Figure S8), which is indicative of the molecular formula C_21_H_35_NO_5_, and is precisely consistent with the anticipated product 16-demethylsaliniketal A [calculated for [M + H]^+^ as 382.2588]. Collectively, based on our biochemical results, we conclude that the Rox enzyme from the Rif producers is the second saliniketal biosynthetic enzyme, which is encoded beyond the Rif biosynthetic gene cluster.

## Discussion

Downstream of the Rif biosynthetic gene cluster from *A. mediterranei* is the *rpoB* gene with mutations in the interaction pocket, which is believed to be the main self-resistance determinant (Floss and Yu, 2005). Another self-resistance mechanism involves the Rif efflux pump RifP, which is regulated by a TetR-family transcriptional regulator RifQ (Lei et al., 2018). In this work, we uncovered a third self-resistance mechanism, that is, the Rox-catalyzed Rif degradation. Interestingly, however, the *Am*Rox gene is transcriptionally silent on the basis of the RNA-seq analysis from the previous report (Shao et al., 2015), which indicates that this gene is a conditionally-activated self-resistance determinant.

Significantly, due to the substrate-promiscuous feature of Rox enzymes, we assigned the second missing saliniketal biosynthetic enzyme. As far as we know, this is the first case that an antibiotic-detoxifying enzyme is simultaneously a designated enzyme involved in another antibiotic biosynthesis. Therefore, saliniketal A is actually biosynthesized by two catalysis-promiscuous enzymes, including the cytochrome P450 (Sare1259 or its counterpart) encoded within the Rif biosynthetic gene cluster that is involved in the biosynthesis of both Rif and saliniketals, and the Rif-degrading monooxygenase Rox. Of the selected Rox proteins, we found that the *Ss*Rox protein from *Streptomyces* sp. TP-A0356 is the most versatile and efficient catalyst with a broad substrate scope, which can be engineered for the generation of saliniketal analogs.

Of note, Koteva et al. (2018) previously used rifabutin and Rif S as competitive inhibitors to assess the Rox-mediated rifampicin monooxygenation and found that the naphthoquinone forms of rifamycins can overcome the monooxygenation reaction. Based on the crystal structures of *Sv*Rox in complex with FAD (and rifampicin) they reasoned that the bulky substituent at position 3 of rifabutin sterically impedes productive binding; although Rif S completely blocked the enzymatic activity, they found it was not a substrate for the monooxygenation reaction (Koteva et al., 2018). In the present study, however, we showed that both 16-demethylrifamycin W and 16-demethylsalinisporamycin of the naphthoquinone form can be accepted by the Rox proteins. Initially, we thought that the two compounds were first spontaneously reduced by NADPH, and then the reduced forms acted as direct substrates. However, we found that the two compounds could not be reduced even under the conditions of a 100-fold excess of NADPH (Supplementary Figure S10a), which led us to investigate Rif S reduction by NADPH. Interestingly, Rif S was easily reduced by NADPH, and converted by *Ss*Rox to the compound **1** (Supplementary Figure S10b). Meanwhile, our time-course analysis of Rif SV degradation revealed that Rif S was produced during the air oxidation of Rif SV and then consumed in the assays (Supplementary Figure S10c). Previous mechanisms of Rif degradation suggest that a C-1 phenolic hydroxyl group is required to trigger the monooxygenation (Koteva et al., 2018; Liu et al., 2018). Specifically, the deprotonation of the C-1 hydroxyl group is not initiated by a catalytic base from Rox but facilitated by the hydrogen bond network between the Rif C-1/C-8 hydroxyl groups and the N5 atom of the reduced FAD cofactor. Next, the resultant anion at C-2 attacks C4a-hydroperoxyflavin, generating the hydroxyl intermediate at C-2, followed by ring-opening to form the degradation product (Supplementary Figure S3). However, for substrates of the naphthoquinone form, the C-2 carbon is actually electrophilic and might be directly attacked by the peroxyflavin to form (hydro)peroxyrifamycin, which is similar to but different from the Baeyer–Villiger oxidation mechanism (Huijbers et al., 2014). The decomposition of (hydro)peroxyrifamycin to the degradation product may be facilitated by the excessive NADPH. This latter mechanism is also consistent with one of the two hypotheses by Wilson et al. (2010; Supplementary Figure S5). Therefore, we believe both mechanisms might well explain the Rif degradation.

In summary, our findings not only demonstrate that the Rox proteins from the Rif producers function as a potential self-resistance determinant to degrade Rif antibiotics, but also complete the gap of the biosynthesis of saliniketal A.

## Materials and Methods

### General

Medium ingredients, biochemicals, and reagents were purchased from Oxoid Ltd (United Kingdom), Sangon Biotech (Shanghai) Co., Ltd (China), Sigma-Aldrich Corporation (United States), and Sinopharm Chemical Reagent Co., Ltd (China), unless otherwise stated. Restriction enzymes and DNA ligases used for gene manipulation were from New England Biolabs (United States), Thermo Fisher Scientific Co., Ltd (United States), and Takara Biotechnology (Dalian) Co., Ltd (China). DNA polymerases used for PCR amplifications were from Vazyme Biotech Co., Ltd (China). PCR primers and chemically synthesized genes were synthesized by GENEWIZ Suzhou (China), and listed in Supplementary Table S1.

### Sequence Analysis of Rox Homologs

As of November 13, 2019, a total of 4,886 protein sequences were retrieved via NCBI BLASTp when using the *N. farcinica* Rox sequence as query under default parameters. These sequences were first clustered via BLASTClust at 80% identity (Alva et al., 2016), and 1,132 representative sequences were obtained. The representative sequences were then used to construct a sequence similarity network under default parameters with an Alignment Score of 110 (Gerlt et al., 2015).

### Protein Expression and Purification

DNA isolation and manipulation in actinomycete strains or *E. coli* were performed according to standard methods (Kieser et al., 2000). PCR amplifications were carried out on a Thermal Cycler (Applied Biosystems). The PCR products were purified (Sangon Biotech) and cloned into the expression vector pET-28a(+) (Novagen).

The resultant recombinant plasmids were introduced into the bacterial expression strain *E. coli* BL21(DE3). When the *E. coli* cultures were grown to an OD_600_ of approximate 0.8 at 37°C in LB medium (800 mL in a 2 L flask, kanamycin 50 μg mL^–1^), the protein expression was induced by adding isopropyl-β-D-thiogalactopyranoside to a final concentration of 0.1 mM, followed by incubation at 16°C for 20 h. For the expression of *Am*Rox, the chaperone plasmid pGro7 (Takara Biotechnology) was required for the assistance of the protein folding and stability.

The cultures were harvested and centrifuged at 1,500 *g* for 10 min. The resultant bacterial pellet was resuspended in 35 mL lysis buffer (50 mM NaH_2_PO_4_, 500 mM NaCl, 10 mM imidazole, and 10% glycerol, pH 8.0), and supersonicated to release the target protein. The *N*-terminally His-tagged protein was purified by the Ni-NTA affinity column (Qiagen). The elution conditions were 30, 10, 10, 4, 4, 4 × 3 mL buffer (100 mM HEPES, 150 mM NaCl, 10% glycerol, pH 7.0) containing 25, 50, 100, 150, 200, and 400 mM imidazole, respectively. Finally, the eluted protein with a yellow color from a buffer containing 400 mM imidazole was desalted using the stock buffer (100 mM HEPES, 150 mM NaCl, 10% glycerol, pH 7.0) by a PD-10 Desalting Column (GE Healthcare). The purified protein was stored at -80°C for subsequent enzymatic assays.

### Enzymatic Assays and Product Analyses

The monooxygenation reaction was carried out in a 200 μL system containing 5–10 μM enzyme, 100 μM substrate, 1 mM NADPH, 100 mM HEPES, and 150 mM NaCl, with a pH 7.0, as described previously (Koteva et al., 2018). The reaction was first incubated at 25°C for 1 h, then quenched by mixing with 200 μL methanol, and finally centrifuged at 13,800 *g* for 5 min. The supernatant was directly used for the HPLC (-MS) analysis.

The HPLC (Agilent Technologies 1260 Infinity) analytic conditions are as follows. The mobile phases A is H_2_O (+0.1% formic acid) and B is acetonitrile (+0.1% formic acid). The gradient condition is 0–2 min 20% B, 2–7 min 20–50% B, 7–9 min 50% B, 9–12 min 50–60% B, 12–14 min 60% B, 14–17 min 60–80% B, 17–19 min 80% B, 19–23 min 80–95% B, 23–28 min 95% B, 28–30 min 95–20% B, and 30–33 min 20% B. The flow rate is 1 mL min^–1^. The analytic column used is Acclaim^TM^ 120 C18, 5 μm, 120 Å, 4.6 × 250 mm.

For the purification of the Rox-catalyzed Rif SV degradation product, the scaled-up reactions were terminated by methanol, followed by centrifugation to precipitate the protein and evaporation to remove the solvent. The compounds were finally dissolved into methanol and directly used for the semi-preparative purification. The semi-preparative gradient condition is 0–3 min 30% B, 3–4 min 30–45% B, 4–7 min 45% B, 7–10 min 45–60% B, 10–12 min 60% B, 12–13 min 60–70% B, 13–14.5 min 70% B, 14.5–15 min 70–95% B, 15–18 min 95% B, 18–19 min 95–30% B, and 19–23 min 30% B. The flow rate is 3 mL min^–1^. The semi-preparative column used is YMC-Pack ODS-AQ 250 × 10.0 mmL.D., S-5 μm, 12 nm.

### Characterization of the Natural Product 16-Demethylsalinisporamycin

The *Micromonospora* sp. TP-A0468 derivative strains were cultured and fermented as previously described (Zhou et al., 2019).

16-demethylsalinisporamycin was purified from the crude fermentation extracts by the silica gel column and Sephadex LH-20 column. HPLC was used to monitor the fractions during the separation process.

High-Resolution ElectroSpray Ionization Mass Spectrometry analyses were performed on the Agilent 6230 Accurate-Mass TOF LC/MS System.

Nuclear magnetic resonance (NMR) data were recorded on the Agilent ProPlus 500 MHz and 600 MHz NMR spectrometers.

## Data AVailability Statement

All datasets generated for this study are included in the article/Supplementary Material.

## Author Contributions

HY, QZ, G-LT, and BX designed the experiments, analyzed the results, and wrote the manuscript. X-FZ performed the biochemical experiments. X-QL and S-YP characterized the compounds.

## Conflict of Interest

The authors declare that the research was conducted in the absence of any commercial or financial relationships that could be construed as a potential conflict of interest.
