# Supplementary material for: Characterization of the Rifamycin-Degrading Monooxygenase From Rifamycin Producers Implicating Its Involvement in Saliniketal Biosynthesis
Source: Front Microbiol. 2020 Jun 3;11:971. doi: 10.3389/fmicb.2020.00971 (PMC7283461; doi:10.3389/fmicb.2020.00971)
Supplement: Supplementary file 1 [file Data_Sheet_1.docx]

Supplementary Material

# Supplementary Table 1 The Rox homologues tested in this study

| Protein | Source | Sequence ID | Primers (5’ to 3’)/chemical synthesis |
| --- | --- | --- | --- |
| *Am*Rox | *Amycolatopsis mediterranei* U32 | YP_003763843.1 | F：TGGTGCCGCGCGGCAGCCATATGGACTCTTCGCCATCCACCACC |
|  |  |  | R：TCGAGTGCGGCCGCAAGCTTCACCGGAGAGGTGAGCCGAACCAG |
| *Sa*Rox | *Salinispora arenicola* | WP_018809167.1 | Chemical synthesis by GENEWIZ |
| *Ms*Rox | *Micromonospora* sp. TP-A0468 | Draft genome in our lab | Chemical synthesis by GENEWIZ |
| *Ss*Rox | *Streptomyces* sp. TP-A0356 | WP_055492794.1 | F：TGGTGCCGCGCGGCAGCCATATGATTGACGTGATCGTTGCC |
|  |  |  | R：TCGAGTGCGGCCGCAAGCTTCAAGTGGTGACGGGCGCGCCGAAC |
| *Nd*Rox | *Nocardioides dokdonensis* | WP_068110870.1 | Chemical synthesis by GENEWIZ |

**Supplementary Figures**

**Supplementary Figure 1.** SDS-PAGE analysis of the tested Rox proteins.

**Supplementary Figure 2.** HR-MS analysis of the compound **1**.

**Supplementary Figure 3**. Rox-catalyzed degradation of rifamycin SV.

| Position | δC | δH | HMBC (H→C) |
| --- | --- | --- | --- |
| 1 |  |  |  |
| 2 | 140.75 |  |  |
| 3 | 106.36 | 5.26 (s, 1H) | 2, 10 |
| 4 |  |  |  |
| 5 | 114.37 |  |  |
| 6 | 171.62 |  |  |
| 7 | 99.31 |  |  |
| 8 | 184.83 |  |  |
| 9 | 106.01 |  |  |
| 10 | 145.64 |  |  |
| 11 | 190.78 |  |  |
| 12 | 105.11 |  |  |
| 13 | 20.52 | 1.61 (s, 3H) | 11, 12 |
| 14 | 7.37 | 1.86 (s, 3H) | 6, 7, 8 |
| 15 | 170.62 |  |  |
| 16 | 130.5 |  |  |
| 17 | 126.45 | 6.02 (d, J = 10.9 Hz, 1H) | 16, 18, 19, 20 |
| 18 | 132.2 | 6.55 (m, 1H) | 16, 20 |
| 19 | 140.75 | 5.76 (dd, J = 15.2, 7.7 Hz, 1H) | 17, 20, 21, 31 |
| 20 | 40.39 | 2.20 (q, J = 7.3 Hz, 1H) | 18, 19, 21, 31 |
| 21 | 73.48 | 3.50 (d, J = 7.8 Hz, 1H) | 19, 20, 23, 32 |
| 22 | 33.94 | 1.75 (s, 1H) | 21, 32 |
| 23 | 75.95 | 3.07 (m, 1H) | 21, 22 |
| 24 | 36.63 | 1.74 (s, 1H) | 23, 33 |
| 25 | 72.56 | 5.24 (s, 1H) | 23, 24, 26, 35 |
| 26 | 40.05 | 1.57 (s, 1H) | 25, 34, 35 |
| 27 | 76.84 | 3.35 (m, 1H) | 25, 26, 28, 29, 34, 37 |
| 28 | 111.6 | 5.10 (dd, J = 11.8, 8.8 Hz, 1H) | 29 |
| 29 | 141.6 | 6.28 (d, J = 12.0 Hz, 1H) | 12, 27, 28 |
| 30 | 20.9 | 1.95 (s, 3H) | 15, 16 |
| 31 | 16.46 | 0.85 (d, J = 7.0 Hz, 3H) | 19, 20, 21 |
| 32 | 10.97 | 0.84 (d, J = 7.0 Hz, 3H) | 22, 23 |
| 33 | 9.82 | 0.72 (d, J = 6.7 Hz, 3H) | 23, 24 |
| 34 | 10.14 | 0.70 (d, J = 6.8 Hz, 3H) | 26, 27 |
| 35 | 182.87 |  |  |
| 36 | 21.89 | 1.57 (s, 3H) | 25, 35 |
| 37 | 55.18 | 2.92 (s, 3H) | 27 |
| 2-OH |  | 15.15 (s, 1H) | 2, 3 |
| 8-OH |  | 11.71 (s, 1H) | 9, 10 |
| 21-OH |  | 4.49 (d, J = 3.0 Hz, 1H) | 20, 21, 22 |
| 23-OH |  | 4.63 (d, J = 6.3 Hz, 1H) | 22, 23, 24 |
| -NH_2_ |  | 7.09 (s, 1H) | 15, 16 |
|  |  | 7.26 (s, 1H) | 15 |

In DMSO-*d_6_*, 600 MHz for ^1^H and 150 MHz for ^13^C NMR.

(a) ^1^H-NMR spectrum

(b) ^13^C-NMR spectrum

(c) DEPT135 spectrum

(d) DEPT90 spectrum

(e) H-H COSY

(f) HSQC

(g) HMBC

**Supplementary Figure 4**. Structural characterization of the compound **1** using NMR.

**Supplementary Figure 5**. Proposed biosynthetic pathway of saliniketal A.

**Supplementary Figure 6**. Characterization of Rox-catalyzed degradation of rifampicin. (a) HPLC analysis of the enzymatic products. The detection wavelength is 324 nm. (b) and (c) HR-MS analyses of rifampicin and the enzymatic product, respectively.

**Supplementary Figure 7**. HR-MS analysis of the compound **3**.

**Supplementary Figure** **8**. HR-MS analyses of 16-demethylsalinisporamycin (a) and 16-demethylsaliniketal A (b), respectively.

| Position | δ_C_ | δ_H_ | HMBC (H→C) |
| --- | --- | --- | --- |
| 1 | 182.87 |  |  |
| 2 | 130.43 |  |  |
| 3 | 115.68 | 7.63 (s, 1H) | 2, 4, 10 |
| 4 | 184.30 |  |  |
| 5 | 106.98 | 7.04 | 4, 7, 9 |
| 6 | 161.67 |  |  |
| 7 | 115.88 |  |  |
| 8 | 163.31 |  |  |
| 9 | 106.94 |  |  |
| 10 | 141.93 |  |  |
|  |  |  |  |
| 12 | 104.30 |  |  |
| 13 | 24.11 | 1.31 (s, 3H) | 12, 29 |
| 14 | 7.91 | 2.04 (s, 3H) | 6, 7, 8 |
| 15 | 166.16 |  |  |
| 16 | 117.87 | 6.3 (d, J=11.3 Hz, 1H) | 15, 18 |
| 17 | 145.07 | 6.64 (dd, J=14.7, 8.3 Hz, 1H) | 15, 19 |
| 18 | 125.95 | 7.44 (dd, J=15.4, 11.3 Hz, 1H) | 15, 20 |
| 19 | 150.69 | 6.25 (dd, J=15.4, 7.8 Hz, 1H) | 17, 20, 21, 31 |
| 20 | 40.83 | 2.37 (m, 1H) | 18, 19, 31 |
| 21 | 73.38 | 3.64 (d, 7.7 Hz, 1H) | 32 |
| 22 | 34.27 | 1.75 (m, 1H) |  |
| 23 | 79.12 | 4.15 (dd, J=6.3, 3.4 Hz, 1H) |  |
| 24 | 33.46 | 1.84 (ddd, J=14.0, 8.9, 5.5 Hz, 1H) |  |
| 25 | 72.81 | 3.83 (d, J=10.5 Hz, 1H) | 26, 27, 34 |
| 26 | 35.73 | 1.7 (t, J=5.8 Hz, 1H) | 27, 28, 34 |
| 27 | 75.56 | 3.35 |  |
| 28 | 23.64 | 1.82 |  |
| 29 | 33.95 | 1.92 |  |
| 31 | 16.22 | 0.92 (d, J=6.5 Hz, 3H) | 20, 21, 22 |
| 32 | 11.15 | 0.93 (d, J=7.0 Hz, 3H) |  |
| 33 | 12.47 | 0.65 (d, J=6.9 Hz, 3H) | 23, 24, 25 |
| 34 | 9.85 | 0.76 (d, J=6.9 Hz, 3H) | 25, 26, 27 |
| CONH |  | 9.84 (s, 1H) | 1, 3, 15 |
| 6-OH |  | 12.11 | 5, 6, 7 |

In DMSO-*d_6_*, 500 MHz for ^1^H and 125 MHz for ^13^C NMR.

(a) ^1^H-NMR spectrum

(b) ^13^C-NMR spectrum

(c) H-H COSY

(d) HSQC

(e) HMBC

**Supplementary Figure 9**. Structural characterization of 16-demethylsalinisporamycin using NMR. Selected ^1^H-^13^C HMBC correlations are illustrated as arrows.

**Supplementary Figure 10**. Reduction of rifamycins of the naphthoquinone form by NADPH (a and b) and time-course analysis of Rif SV degradation using *Ss*Rox by HPLC (c). In the reaction system, the concentration of Rif SV/Rif S is 100 µM, while the concentration of NADPH is 1 or 10 mM. Rif SV is easily oxidized to Rif S in the air; and Rif S is easily reduced to Rif SV by NADPH. The intermediate compound * is proposed to be transformed to the product **1**.
